# Paediatric injuries at Bugando Medical Centre in Northwestern Tanzania: a prospective review of 150 cases

**DOI:** 10.1186/1752-2897-7-10

**Published:** 2013-11-13

**Authors:** Raymond Simon, Japhet M Gilyoma, Ramesh M Dass, Mabula D Mchembe, Phillipo L Chalya

**Affiliations:** 1 Department of Surgery, Catholic University of Health and Allied Sciences – Bugando, Mwanza, Tanzania; 2 Department of Orthopaedic & Trauma, Catholic University of Health and Allied Sciences – Bugando, Mwanza, Tanzania; 3 Department of Surgery, Muhimbili University of Health and Allied Sciences, Dar Es Salaam, Tanzania

**Keywords:** Paediatric injuries, Etiological spectrum, Injury characteristics, Treatment outcome, Tanzania

## Abstract

**Background:**

Injuries continue to be the leading cause of death and disability for children. The is a paucity of published data on paediatric injuries in our local environment. This study describes the etiological spectrum, injury characteristics and treatment outcome of paediatric injuries in our local setting and provides baseline data for establishment of prevention strategies as well as treatment guidelines.

**Methods:**

This was a descriptive cross-sectional study involving paediatric injury patients admitted to Bugando Medical Centre from August 2011 to April 2012. Statistical data analysis was done using SPSS version 17.0 and STATA version 12.0.

**Results:**

A total of 150 patients were studied. The age of patients ranged from 1 month to 10 years with a median age of 5 years. The male to female ratio was 2.3:1. Road traffic accident was the most common cause of injury (39.3%) and motorcycle (71.2%) was responsible for the majority of road traffic accidents. Only 11 (7.3%) patients received pre-hospital care. The head /neck (32.7%) and musculoskeletal (28.0%) were the most frequent body region injured. Open wounds (51.4%), foreign bodies (31.3%) and fractures (17.3%) were the most common type of injuries sustained. The majority of patients 84 (56.0%) were treated surgically. Complication rate was 3.9%. The mean duration of hospitalization was 9.7 ± 13.1 days. Mortality rate was 12.7%. Age of the patient (< 5 years), late presentation and presence of complications were the main predictors of length of hospital stay (P < 0.001), whereas burn injuries, severe head injuries and severity of injury (Paediatric trauma score = 0–5) significantly predicted mortality (P < 0.0001).

**Conclusion:**

Paediatric injuries resulting from road traffic accidents (RTAs) remain a major public health problem in this part of Tanzania. Urgent preventive measures targeting at reducing the occurrence of RTAs is necessary to reduce the incidence of paediatric injuries in this region.

## Background

Trauma is reported to be an important cause of childhood morbidity, and mortality in developed countries while causing an increasing loss of life in developing countries [1]. In the United States, over 1.5 million childhood traumas occur annually, resulting in approximately 600 000 hospitalizations and 15 000–20 000 paediatric deaths each year [2]. In the European region, injuries account for 23% of deaths from all causes and 19% of disability-adjusted life years (DALYs) from all causes in the age group 0–19 years [3]. In Africa, the true incidence is not known but injuries have been estimated to account for 13% of childhood disease burden and nearly 1million deaths occur per year in developing countries, including Africa [4,5].

In Tanzania, like other developing countries, injuries constitute a major but neglected public health problem and yet have a significant adverse effect on the country’s economy and health services in terms of morbidity, mortality and long term disability among paediatric population [6]. Paediatric injuries are a single commonest cause of paediatric surgical admissions at Bugando Medical Centre and contribute significantly to high morbidity and mortality.

With increasing motorization and criminal activities in both urban and semi-urban communities of developing countries, the incidence of traumatic injuries in children is on the increase [7]. Children aged 10 years and below are particularly at risks for injuries because they are unable to recognize and avoid many potential risks for injuries due to their low level of judgment exposing them to great danger of accidents [7,8].

The causes and pattern of paediatric injuries have been reported to vary according to geographic area, socio-economic status and environment factors [9]. The purpose of studying injury characteristics and its causes is to establish programmes to prevent and control its development and spread [7,10].

It has been shown that improved hospital care results in lower mortality and that care is best delivered at a paediatric trauma centre [11]. Therefore the identification of high-risk injury patterns may lead to improved care and ultimately further improvements in outcome in children admitted to hospital with trauma [12].

Since the majority of paediatric injuries are preventable, a clearer understanding of the causes, injury patterns and outcome of these patients is essential for establishment of prevention strategies as well as treatment protocols [7,13].

There is paucity of data on paediatric injuries in Tanzania and the study setting in particular. Such information is necessary for assessing the impact of trauma on child health and for setting priorities to improve paediatric trauma care. The aim of this study was to outline the etiological spectrum, injury characteristics and outcome of paediatric injuries and to identify the predictors of the outcome of these patients in our setting. The study results will provide basis for planning of prevention strategies and establishment of treatment protocols.

## Methods

### Study design and setting

This was a descriptive prospective study involving paediatric injury patients admitted to Bugando Medical Centre (BMC) over a nine-month period from August 2011 to April 2012 inclusive.

The study was conducted at the A & E department of Bugando Medical Centre. BMC is one of the four largest referral hospitals in the country and it is located in Mwanza city in the northwestern part of Tanzania. It has a bed capacity of 1000 and serves as a referral centre for tertiary specialist care for a catchment population of approximately 13 million people from Mwanza, Mara, Kagera, Shinyanga, Tabora and Kigoma. It is also a consultancy and teaching hospital for the Catholic University of Health and Allied Sciences-Bugando (CUHAS-Bugando) and other paramedics. Paediatric injury patients are first seen at the A & E department where the surgical team does primary and secondary surveys according to advanced trauma life support (ATLS). From the A & E department these patients are either discharged home or admitted in pediatric surgical wards after definitive treatment either in operating theatre or at the A & E department. Depending on the severity of injury, patients may also be admitted to the intensive care unit (ICU).

### Study subjects (patients)

The study included all pediatric injury patients aged 10 years and below presenting to the A & E department and paediatric surgical ward of BMC during the study period. Patients without next of kin to consent for the study and those who died before complete assessment were excluded from the study. Convenience sampling of patients who met the inclusion criteria was performed until the sample size is reached. Recruitment of patient to participate in the study was done at the A & E department after primary and secondary surveys done by the admitting surgical team. Patients were screened for inclusion criteria and those who met the inclusion criteria were offered explanations about the study and requested to consent before being enrolled into the study. All recruited patients were first resuscitated in the A & E department according to the Advanced.

Trauma Life Support (ATLS) principles and were then taken into the paediatric surgical ward or the intensive care unit (ICU) from where necessary investigations were completed and further treatment was instituted.

The severity of injury was determined using the Paediatric trauma score (PTS) [14]. Severe injury consisted of a PTS 0–5, moderate injury 6–8, and mild injury 9–12. Patients with head injuries were classified according to Glasgow Coma Scale (GCS) into: severe (GCS 3–8), moderate (GCS 9–12) and mild (GCS 13–15). Depending on the type of injury, the patients were treated either conservatively or by surgery.

Patients were followed up till discharge or death. Data were collected using a pre-tested coded questionnaire. Data administered in the questionnaire included; demographic characteristics (e.g. age, sex), circumstances of injury, characteristics of injury, treatment modalities, Length of Hospital Stay (LOS) and mortality.

### Statistical data analysis

Statistical data analysis was done using SPSS software version 17.0 and STATA version 12.0. Data were summarized in form of proportions, frequent tables, bar and pie charts for categorical variables. Means, median, mode, standard deviation and histograms were used to summarize continuous variables. Chi-square test was used to test for significance of associations between the predictor and outcome variables in the categorical variables. Student t-test was used to test for significance of associations between the predictor and outcome variables in the continuous variables. Significance was defined as a p-value of less than 0.05. Multivariate logistic regression analysis was used to determine predictor variables that are associated with outcome.

### Ethical consideration

Ethical approval to conduct the study was obtained from the CUHAS-Bugando/BMC joint institutional ethic review committee before the commencement of the study. Informed consent was sought from each patient’s next of kin/parents before being enrolled into the study.

## Results

### Patient’s characteristics

**Figure 1:**
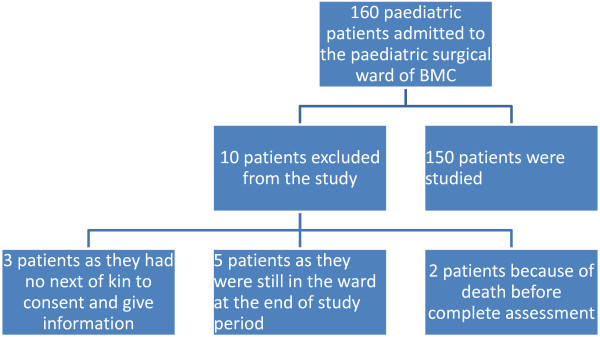
**Flow chart of patients showing number of patients.** Keys: BMC = Bugando Medical Centre.

During the period under review, a total of 160 paediatric injury patients were admitted to the paediatric surgical ward. Of these, 10 patients were excluded from the study due to failure to meet the inclusion criteria. Thus, 150 patients were studied (Figure 1). The age of patients ranged from 1 month to 10 years with a median of 5 years. The peak age incidence was 6–8 years. One hundred and four (69.3%) were males and 46 (30.7%) were females. the male to female ratio of 2.3: 1 with a male predominance in each age group. In this study, no patient had premorbid illness.

### Circumstances of injury

Regarding the time of injury, 125 (83.3%) patients sustained injury during the day, 22 (14.7%) at night and in 3 (2.0%), the time was not specified. Most of injuries occurred at home (57.3%) as shown in Table 1. All patients in this study sustained unintentional injuries. Road traffic accident was the most common cause of injury accounting for 39.3% of cases (Table 1). Motorcycle (42, 71.2%) was responsible for the majority of road traffic accidents, followed by motor-vehicles in 17 (28.8%) patients. Pedestrians (50, 84.7%) accounted for the majority of victims, followed by passengers (9, 15.3%). Most patients (118, 78.7%) in this study sustained blunt injuries. Other mechanisms of injury included burn and foreign body inhalation/ingestion in 32(21.3%) and 47 (31.3%) patients respectively. The vast majority of patients (126, 84.0%) reported to the A & E department within 24 hours after injury.

**Table 1:** Distribution of patients according to the circumstances and characteristic of injury

**Variables**	**Number of patients**	**Percentage**
**Time of injury**		
Day	125	83.3
Night	22	14.7
Time not specified	3	2.0
**Place of injury**		
Home	86	57.3
Sideway/along road	59	39.3
Recreation	3	2.0
School	2	1.3
**Nature of injury**		
Intentional	0	0
Unintentional	150	100
**Cause of injury**		
RTA	59	39.3
Falls	12	8
Burn	32	21.3
Foreign body	47	31.3
**Mechanism of injury**		
Blunt	71	47.3
Burn	32	21.3
Foreign body ingestion	47	31.3
**Arrival time**		
≤24 hrs	124	83
>24 hrs	26	17
**Pre hospital care**		
Done	11	7.3
Not done	139	92.7
**Waiting time**		
≤6 hrs	125	83.3
6 – 24 hrs	24	16.0
>24 hrs	1	0.67
**Body region affected**		
Head/neck	49	32.7
Chest	20	13.3
Abdomen/pelvic	5	3.3
Extremities	42	28.0
Multiple body part	34	22.7

In this study, only 11 (7.3%) patients received pre-hospital care. The majority of (111, 74.0%) were brought in by relatives/friends or Good Samaritans. 32 (21.3%) were brought in by ambulance and 7 (4.7%) were brought in by police. The means of transport from the site of injury to hospital in the majority of patients was private transport in 80 (72.1%), public transport in 24 (21.6%) and motorcycle in 7 (6.3%) patients.

The majority of patients (125, 83.3%) were attended to within 6 hours of arrival to the A & E department as shown in Table 1.

### Injury characteristics

The head/neck and musculoskeletal (extremities) were the most frequent body region injured accounting for 32.7% and 28.0% of cases respectively (Table 1). Isolated injuries occurred in 116 (77.3%) patients while 34 (22.7%) patients had multiple injuries. Open wounds (i.e. bruises, abrasions, lacerations, cut wounds, burn wounds etc.), foreign bodies and fractures were the most common type of injuries sustained (Table 2). In patients with burn injuries, scald was the most common type of burn in 24 (84.4%) patients followed by flame burn in 5 (15.6%) patients. There was no chemical, electrical or radiation burns. The % TBSA among burn injury patients ranged from 5-50% with a median of 14.0%.

**Table 2:** Distribution of patients according to the type of injury

**Type of injury**	**Frequency**	**Percentage**
Bruises	45	30.0
Laceration	9	6.0
Cut wounds	12	8.0
Burn wounds	32	21.3
Fractures	26	17.3
Sprain	13	8.7
Dislocation	9	6.0
Foreign bodies	47	31.3

According to Paediatric Trauma Score (KTS), the majority of patients sustained mild injuries (PTS = 9–12) in 85 (56.7%) patients. Moderate injuries (PTS = 6–8) and severe injuries (PTS = 0–5) were recorded in 60 (40.0%) and 5 (3.3%) patients respectively. The PTS ranged from 5 to12 with a median of 9.0. The Glasgow coma scale indicated that most of the patients (15, 51.7%) sustained moderate head injury, 8 (27.6%) patients sustained severe head injury and 6 (20.7%) patients had mild head injury.

### Admission pattern and treatment modalities

A total of 140 (93.3%) patients were admitted in the paediatric surgical wards and the remaining 10 (6.7%) patients were admitted to the intensive care unit (ICU). The majority of patients 84 (56.0%) were treated surgically as shown in Table 3.

**Table 3:** Distribution of patients according to surgical procedure performed

**Surgical procedure performed**	**Frequency**	**Percentage**
Wound debridement	21	14.0
Treatment of fractures	26	17.3
Bronchoscopy ± foreign body removal	14	9.3
Esophagoscopy ± foreign body removal	32	21.3
Skin grafting	5	3.3
Amputation	2	1.3
Craniotomy	2	1.3
Margil’s foreign body removal	1	0.7

### Treatment outcome

Five patients developed complications giving a complication rate of 3.9%. Wound sepsis was the most common complications in 3 (2.0%) patients followed by post-burn contracture and pneumonia in 1 (0.7%) patient each respectively. The overall LOS ranged from 1 to 72 days with a mean and median of 9.7 ± 13.1 days and 4.0 days respectively. The mode was 2.0 days. The LOS for non-survivors ranged from 1 day to 16 days with a mean of 2.6 ±6.3 days. The median and the mode were 1.0 day each respectively.

Table 4 shows predictors of LOS according to univariate analysis and multivariate logistic regression analysis. In this study, nineteen patients died giving a mortality rate of 12.7%. Table 5 shows predictors of mortality according to univariate analysis and multivariate logistic regression analysis.

**Table 4:** Predictors of LOS according to univariate analysis and multivariate logistic regression analysis

**Independent variables**	**Univariate analysis**				**Multivariate analysis**	
	**≤ 14 days**	**>14 days**	**OR [95% CI]**	**P -Value**	**OR 95% CI**	**P -Value**
**Age group**						
<5 yrs	44 (62.9)	26 (37.1)	3.3[1.5-7.3]	0.002	3.9[1.60-9.4]	0.003
5 – 10 yrs	68 (85)	12 (15)	1			
**Sex**						
M	77 (74.0)	27 (26.0)	1			
F	35 (76.1)	11 (23.9)	0.9[0.4-2.0]	0.790		
**Injury arrival time**						
≤ 24 hrs	93 (73.8)	33 (26.2)	1		0.2[0.04-0.91]	0.037
> 24 hrs	19 (79.2)	5 (20.8)	0.7[0.26-2.15]	0.581		
**Pre hosp care**						
Done	6 (54.6)	5 (45.5)	1			
Not done	106 (76.3)	33 (23.7)	0.4[0.11-1.3]	0.122		
**Mech injury**						
Blunt	101(85.6)	17(14.4)	1			
Burn	11(34.4)	21(65.6)	11.3[4.6-27.7]	<0.001		
**Wait time**						
< 6 hrs	93(74.4)	32(25.6)	1			
≥ 6	19(76.0)	6 (24.0)	0.9[0.34-2.5]	0.867		
**PTS**						
Mild	70(82.3)	15(17.7)	1			
Mode/severe	42(64.6)	23(35.4)	2.6[1.2-5.4]	0.015		
**Complications**						
Yes	1(20.0)	4(80.0)	1			
No	92(74.8)	31(25.2)	0.5[0.2-0.8]	0.008	1.5[1.2-6.4]	0.014

**Table 5:** Predictors of mortality according to univariate analysis and multivariate logistic regression analysis

**Independent variables**	**Alive**	**Dead**	**Univariate analysis**		**Multivariate analysis**	
	**N = 131**	**N = 19**	**OR (95% CI**	**P -value**	**OR (95% CI)**	**P- Value**
**Age group**						
≤5	63(90)	7(10)	1			
5- 10	68(85)	12(15)	1.6[0.6-4.3]	0.361		
**Sex**						
M	93(89.4)	11(10.6)	1			
F	38(82.6)	8(17.4)	1.8[0.7-4.8]	0.252		
**Arriving time**						
≤24 hrs	112(88.9)	14(11.1)	1			
>24 hrs	19(79.2)	5 (20.8)	2.1[0.7-6.5]	0.197		
**Prehospital care**						
Not done	124(89.2)	15(10.8)	1			
Done	7(63.6)	4(36.4)	4.7[1.2-18.0]	0.023		
**Mechanism of injury**						
Blunt	108(91.5)	10(8.5)	1			
Burn	23(71.9)	9(28.1)	4.2[1.5-11.6]	0.005	38.8[2–750.9]	0.016
**Waiting time**						
≤6 hrs	107(85.6)	18(14.4)	1			
>6 hrs	24(96)	1 (4.0)	0.25[0.3-1.9]	0.185		
**Affected part**						
Trunk/extremities	66(98.5)	1 (1.5)	1			
Head	40(81.6)	9(18.4)	14.6[1.8-121.6]	0.012	284[12.3-6592.6]	0.000
Multiple	25(73.5)	9 (26.50	23.8[2.7-197.3]	0.003	27[2.6-285.1]	0.006
**PTS**						
Mild	83(97.6)	2(2.4)	1			
Moderate/severe	48(73.8)	17(26.2)	7.2 [3.6- 11.8]	0.012	3.9 [3.1-5.9]	0.000
**GCS**						
Mild	117(96.7)	4(3.3)	1			
Moderate/severe	14(48.3)	15(51.7)	2.6 [1.4 – 9.9]	0.022	3.8 [2.5-34.4]	0.003

## Discussion

### Patient’s characteristics

Children have a unique profile of risks for injuries because they are unable to recognize and avoid many potential risks on their own [15,16]. In this study, the peak age incidence was 6–8 years which is in agreement with other studies done elsewhere [17,18]. High incidence of injuries in this age group reflects lack of coordination and unawareness of dangerous substances. In addition, this is the school-age group and is usually involved in road traffic accidents as they rush through heavy traffic to and from their schools. These school-age group children are usually very active and are often less supervised than pre-school age children. This observation calls for an improved school transportation system.

In our study, males were more affected than females with a male to female ratio of 2.3:1 which is in agreement with other studies [19]. The reasons for the male preponderance in our study may be attributed to the overactive nature of male children as compared to the females.

The presence of pre-existing illness has been reported to have an impact on the outcome of paediatric injury patients [20]. In the present study, no patient had pre-existing illness.

### Circumstances of injury

With regard to the time injury, most of injuries in the present study occurred during the day which is in agreement with that of other studies [17,21]. Increased rate of injuries during the day can be explained by increased traffic jams as well as increased human activities in the city during the day time. Knowing the time of injury in trauma patient is important for prevention strategies.

The majority of paediatric injury in this study occurred at home, which is in agreement with other studies done elsewhere [21,22]. This finding is at variant with an Iranian study which reported streets as the most common place of occurrence of paediatric injuries [17]. The finding that most of paediatric injuries occurred at home demonstrates the important role of parental supervision as a key factor in child safety.

In this study, all paediatric injury patients sustained unintentional injuries resulting from road traffic accidents, falls, burns and foreign body inhalation/ingestion. There were no cases of intentional injuries. However, the lack of intentional injuries in our study may actually be an underestimate and the magnitude of the problem may not be apparent because many cases are not reported for fear of been arrested by police. Therefore, paediatric forensic examination should be performed if a child is likely to suffer from abuse, neglect or intentional injury.

Road traffic accidents have been reported to be the commonest cause of blunt paediatric injuries in most studies as supported by the present study [23]. In contrast to our findings, a study in Malawi reported fall from height as the most common cause of paediatric injuries [24]. A study in Kenya reported that burn injuries as the most frequent cause of paediatric injuries [18]. High incidence of road traffic accidents in our study may be attributed to recklessness and negligence of the driver, poor maintenance of vehicles, driving under the influence of alcohol or drugs and complete disregard of traffic laws. Improvement in road conditions, prevention of overloading of commuter vehicles, maintenance of vehicles and encouraging enforcement of traffic laws will decrease the frequency and extent of these injuries. In agreement with other studies [25,26], motorcycle (71.2%) was responsible for the majority of road traffic accidents. The prevalence of motorcycle injuries in this study is higher than that reported previously at the same centre by Chalya *et al.*[26] reflecting increase in the magnitude of the problem in our setting. Motorcycle use is becoming popular in Tanzania as it has become a cheaper and easier means of transportation in most cities. However their use is characterized by non-helmet use by riders and their passengers, passenger overload, lack of certified driver training and valid licensing, over speed and reckless driving, poor regulation and law enforcement and possible use of alcohol and drugs. In this study, pedestrians (84.7%) accounted for the majority of road traffic victims, which is in keeping with other study done elsewhere [17]. High incidence of pedestrians among children has been attributed to their developmental and behavior limitations in complex traffic situations [10,11]. Pedestrians aged 10 years and below are particularly vulnerable because of their small physical size and underdeveloped abilities to dealing with traffic situations, both cognitive (attention focus, interpreting signs) and perceptual (locating sounds, judging speed, peripheral vision) [11]. Children under the age of 10 years do not have the ability to cross roads without adult help.

Injuries related to foreign bodies in the aerodigestive tract was the second most common cause of paediatric injuries in our locality as previously reported by Gilyoma and Chalya [27] at the same centre. In agreement with other studies [27-29], our study found that foreign bodies in the esophagus was more prevalent than in the bronchus, ear or nose. In this study, we could not establish the reason for this anatomical distribution. Several factors contribute to high incidence of aerodigestive tract foreign bodies in this age group including social factors (e.g. carelessness of parents, children’s habit of putting objects in their mouth, crying/playing during eating) and anatomical factors (e.g. absent of molar teeth, inadequate control of deglutition) have been mentioned in literature [30,31].

The prehospital care of injured paediatric patients is the most important factor in determining the ultimate outcome after injury [12]. In our study, only 7.3% of patients had pre-hospital care. The lack of advanced pre-hospital care in most developing countries like Tanzania and ineffective ambulance system for transportation of patients to hospitals are a major challenges in providing care for paediatric injury patients in these countries and have contributed significantly to poor outcome of these patients due to delay in definitive treatment.

The majority of patients in our study reported to the A & E department within 24 hours of injury, which is in keeping with other reports [17,21]. Our experience shows that early presentation is common with very young children, and when there are more serious symptoms of severe injury, thus compelling the frightened patients or parents to seek medical attention. Late presentation is more common in asymptomatic and mild cases. Gilyoma and Chalya [27] in their experiences with endoscopic procedures for removal of foreign bodies of the aerodigestive tract at Bugando Medical Centre found that the majority of patients mainly children presented to the A & E department within 24 hours of inhalation/ingestion of foreign.

Waiting time in emergency departments may be attributable to many factors and may stretch up to three hours before completion of all necessary procedures, even in developed countries [32]. This study found that the majority of the patients (83.3%) were attended to within 6 hours of arrival at the A & E Department. Lambe *et al*. [33] reported a lower mean waiting time of 56 minutes in California, USA. Review of emergency department administration has been demonstrated to improve efficiency in care delivery [34]. A waiting time of 30 minutes for a general outpatient clinic is considered reasonable but should be even shorter for emergency visits [35].

### Injury characteristics

In agreement with previous studies [17,18], the present study found that the head and the musculoskeletal (extremities) were the most common body region injured and the former accounted for most of the deaths and admission to intensive care. Higher incidence of head injuries in most previous studies as well as our study may be attributed to the disproportionately large head and weak neck musculature in children that puts them at particular risk for contre-coup brain injuries even at low velocity injury [21]. Our high figure of musculoskeletal injuries affecting mainly the lower limbs is attributable to the large number of pedestrians. Pedestrians are unprotected road users and therefore they are highly exposed to high risk of limb injuries [36].

The type of injuries in this study is comparable with what is reported in other studies [17,21,36]. In the present study, open wounds (i.e. bruises, abrasions, lacerations, cut wounds, burn wounds etc.), foreign bodies and fractures were the most common type of injuries sustained.

A number of scoring systems have been developed to facilitate consistent trauma triage, severity evaluation, management and prognostication [37]. Paediatric Trauma Score (PTS) is one of trauma scores designed to accurately assess injury severity and extent of injury, aid with the prediction of survival and subsequent morbidity [38]. The PTS was devised specifically for the triage of paediatric trauma patients [14]. The PTS is calculated as the sum of individual scores from six clinical variables including weight, airway, systolic blood pressure (SBP), central nervous system (CNS) status (level of consciousness), presence of an open wound, and skeletal injuries [39,40]. According to Paediatric Trauma Score (PTS), the majority of patients in this study sustained mild injuries accounting for 56.7% of cases. The Glasgow coma scale (GCS) was developed as a means of assessing a patient’s level of consciousness by assigning coded values for three behavioral responses [14,39,40]. In this study, the GCS indicated that most of the patients sustained moderate head injury.

### Treatment modalities

Most of our patients were treated surgically, which is in agreement with other similar studies [41,42]. The high incidence of surgical treatment in our study is attributable to the high incidence of injuries that required surgical intervention. In this study, endoscopic removal of aerodigestive foreign bodies, treatment of fractures and wound debridement were the most frequent surgical procedure performed. Regarding endoscopic removal of aerodigestive foreign bodies, oesophagoscopy for removal of foreign bodies was the most common procedure performed as reported earlier by Gilyoma and Chalya [27].

### Treatment outcome

The presence of complications has an impact on the final outcome of patients presenting with paediatric injuries as supported by the present study [43,44]. The pattern of complications in the present study is similar to what was reported by others [17,23,36]. Early recognition and management of complications following paediatric injury is of paramount in reducing the morbidity and mortality resulting from this form of trauma.

The length of hospital stay (LOS) has been reported to be an important measure of morbidity among trauma patients and has an impact on patient’s final outcome [43,44]. Prolonged duration of hospital stay is associated with unacceptable burden on hospital resources as well as on increased costs of health care [45]. In the present study, the overall average LOS in the present study was higher than that reported by others [3,21]. The reasons for prolonged LOS in our study according to multivariate logistic regression analysis included young patient’s age, delayed presentation and presence of complications.

The current study had a mortality rate of 12.7%, which is higher than that reported by others [46-48]. Factors responsible for high mortality in our study included burn injuries, severe head injuries, severe injuries and multiple injuries. Addressing these factors responsible for high mortality in our patients is mandatory to be able to reduce mortality associated with these injuries.

## Conclusion

Paediatric injuries resulting from road traffic accidents (RTAs) remain a major public health problem and contribute a substantial proportion of all paediatric surgical admissions at Bugando Medical Centre. Most of injuries occurred at home and sideways/along roads. Head and musculoskeletal injuries were the most common body region injured predisposing these patients to prolonged hospitalization and mortality. Open wounds (i.e. bruises, abrasions, lacerations, cut wounds, burn wounds etc.), foreign bodies and fractures were the most common type of injuries sustained. It is therefore recommended that:-

•Urgent preventive measures targeting at reducing the occurrence of RTAs is necessary to reduce the incidence of paediatric injuries in this region.

•The finding that most of paediatric injuries occurred at home and sideways/along roads calls for a need for educational and intervention programmes to increase the awareness and understanding of child safety and injury prevention in our local setting and to make the home a safe environment for children.

•Advanced pre-hospital care and effective ambulance system for transportation of patient to hospital is highly needed in our setting in order to improve the outcome of these patients.

•Early presentation to the hospital for early diagnosis and definitive treatment following these injuries is highly recommended to reduce the morbidity and mortality resulting from such injuries.

## Competing interests

The authors declare that they have no competing interests.

## Authors’ contributions

RS–Study design, data analysis, manuscript writing & editing, JMG, RMD, MDM participated in data analysis, manuscript writing & editing and PLC participated in data analysis, manuscript writing, final editing and submission of the manuscript. All authors read and approved the final manuscript.
